# Safety in Marriage

**Published:** 1907-02

**Authors:** 


### SAFETY IN MARRIAGE.

A. H. Burr, of Chicago, pleads for sanitary regulations that will guarantee the safety of woman in the marriage contract. The prevalence of venerial disease, especially gonorrhea, which is the cause of the ill-health of so many innocent women, demands special protective legislation. "To insure a sanitary marriage it is imperative to establish a quarantine station before the marriage license window over whose gate should hang this legend: 'No health Certificate, No License.'" He commends the North Dakota law which requires all applicants for the marriage license to present a certificate from a medical examining board of three physicians, appointed by the county judge, showing freedom from venereal disease, habitual drunkenness, insanity and tuberculosis. He appeals to physicians to give their influence and support for the enactment of satisfactory laws to restrict and to suppress the contagious perils of venery. Only by this can we count on a reasonable guarantee of safety to woman in the marriage contract.—*Jour. A. M. A.*, December 8, 1906.

